# Outcomes and patterns of treatment in chronic myeloid leukemia, a global perspective based on a real-world data global network

**DOI:** 10.1038/s41408-022-00692-8

**Published:** 2022-06-24

**Authors:** A. Sanz, R. Ayala, G. Hernández, N. Lopez, D. Gil-Alos, R. Gil, R. Colmenares, G. Carreño-Tarragona, J. Sánchez-Pina, R. A. Alonso, N. García-Barrio, D. Pérez-Rey, L. Meloni, M. Calbacho, J. Cruz-Rojo, M. Pedrera-Jiménez, P. Serrano-Balazote, J. de la Cruz, J. Martínez-López

**Affiliations:** 1 Hematology Department, Hospital 12 de Octubre, Complutense University, CNIO, Madrid, Spain; 2 Biomedical Informatics Group, Universidad Politécnica de Madrid, Madrid, Spain; 3 Data Science Group, Research Institute imas12, Hospital 12 de Octubre, Madrid, Spain; 4 TriNetX, LLC, Cambridge, MA USA; 5 Research Institute imas12, Hospital 12 de Octubre, Madrid, Spain

**Keywords:** Chronic myeloid leukaemia, Epidemiology

The natural history of chronic myeloid leukemia (CML) experienced a major change in the early 2000s with the development of the first BCR-ABL1 specific tyrosine kinase inhibitors (TKIs) [1, 2]; however, there is limited supporting evidence from electronic health records (i.e., real-world data [RWD]). TriNetX is a global health research platform that provides researchers access to electronic medical records from healthcare organizations (HCOs) for conducting research studies. Our goal was to better define the current survival landscape of CML and the long-term toxicities so that we can have a better and updated understanding of the disease and the actual clinical practice in local, regional, and global settings.

This study was conducted with anonymized data accessed via the TriNetX platform, which has already proven to be useful in performing real-world data studies that are helpful in clinical practice [3–5]. Data from approximately 6000 CML patients treated with TKIs from 57 HCOs were obtained via TriNetX. Hospital 12 de Octubre (H12O) participates in this platform through its novel methodology for the effective reuse of the EHR [6]. Thus, the analyses were run on four different patients cohorts to independently confirm our hypotheses: H12O, with around 1,000,000 patients; the EMEA Collaborative Network (EMEA), with 11,000,000 patients from 18 HCOs; the US Collaborative Network (US), with 73,000,000 patients from 47 HCOs, and the Global Collaborative Network (Global), with 90,000,000 patients from 75 HCOs. All data collection, processing, and transmission were done in compliance with all data protection laws applicable to the contributing HCOs. Analytics are performed at the HCO with only aggregate results being returned to the platform. Individual personal data does not leave the HCO. From these main study populations, we constructed several cohorts (Fig. 1) so we could make all necessary comparisons required for the study. All analyses were conducted on December 2021 using the analytics built into the TriNetX platform.

**Fig. 1:**
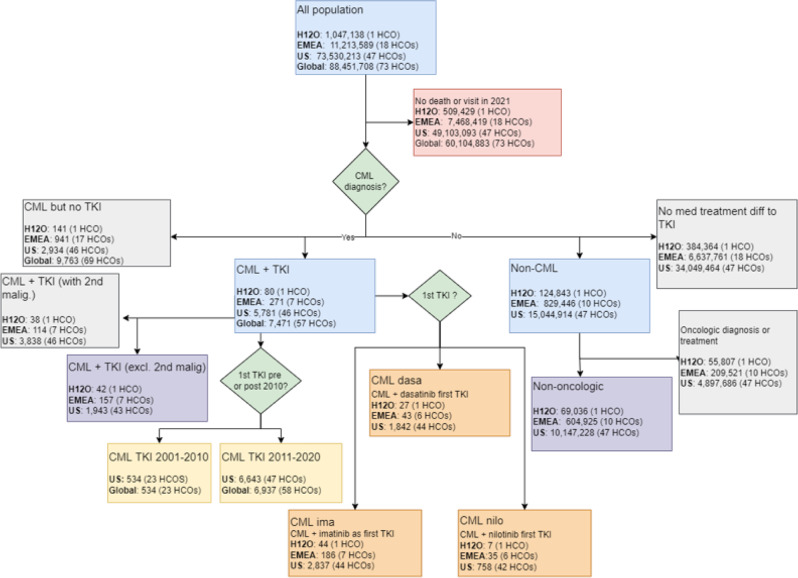
Cohorts construction from the main study populations. First, to identify the adverse event profile associated with CML, we matched (1:1) CML patients in the H12O, EMEA, and U.S. networks to non-CML patients from the same network using propensity score matching based on age and sex. Second, for the analysis that assesses the effect of CML on the survival and infection (ICD-10-CM diagnosis branch A00-B99) incidence of patients, we compared non-oncologic patients from the H12O, EMEA, and U.S. networks to the subset of CML patients from the same network without any other neoplasm, i.e., ICD-10-CM diagnosis in the branch C00-D49 (Neoplasms) other than C92.1 using propensity score matching (1:1) based on age and sex. Third, sub-cohorts from the original CML cohorts from the H12O, EMEA, and U.S. networks, based on the first TKI record in the EMR, were created to compare survival and incidence of second malignancy between patients who received imatinib as their first TKI versus those who received dasatinib or nilotinib. Fourth, we segmented the original CML cohorts from the United States and Global networks into two cohorts based on whether they received their first TKI between 2001 and 2010 or between 2011 and 2020.

The total number of CML patients in the study period was 80 in H120, 271 in the EMEA, and 5782 in the United States. The frequency of new cases in the analyzed population was mostly stable, ranging from 8·4 to 15·1 cases per 100,000 patients per year (mean: 12·3) (Supplementary Fig. 1A, B, C). Analyzing the proportion of patients receiving each TKI as first-line of treatment and the frequency of subsequent switching to other TKIs (Supplementary Fig. 1A, B, C, exact counts in Supplementary Table 1), imatinib was the most used TKI as first-line treatment in all cohorts, and dasatinib the most used as second-line.

We then analyzed differences in the development of other cancers, cardiovascular diseases, and relevant infectious diseases after TKI initiation. The compared cohorts in each geographical group were CML patients vs non-CML patients. Applying propensity score matching for age and gender, the final cohort sizes were 57 in H12O, 247 in the EMEA, and 5249 in the United States (Supplementary Table 2). The H12O shows a higher incidence of second malignancies in the CML group (43·9 vs 28·1%), (*p* = 0·079). The EMEA cohorts show the same tendency (40·5 vs 20·6%, *p* < 0·001), and the difference was statistically significant (OR 2.61 with 95% CI (1·75, 3·89)). In the U.S. cohorts, out of 5249 patients, 3380 (64·4%) CML patients developed a second malignancy, compared to only 902 (17.2%, *p* < 0·001) of the non-CML cohort (OR 8·72 with 95% CI (7·96, 9·55)). This risk is considerably higher than previously reported [7–9]. It is likely that this phenomenon is inherent to the person already having a neoplastic condition and not truly related to the TKI treatment, since no TKI showed a clear higher risk than the others.

Regarding cardiovascular diseases, in EMEA there was a statistically significant higher incidence of cardiovascular disease in the CML cohort (64·4 vs 51·8%, *p* < 0·005 with OR 1·68 95% CI (1·17, 2·41). This was also observed in the U.S. cohort, despite higher rates of cardiovascular disease overall than in H12O and EMEA cohorts (74·2 vs 60·5%, *p* < 0·001, OR 1·88 (1·73, 2·04)). No significant difference was found when analyzing if the occurrence of cardiovascular diseases differed among patients receiving imatinib, dasatinib, or nilotinib as first-line treatment. However, pleural effusion had a clear higher incidence with dasatinib (OR 2·27 (1·76, 2·92) compared with nilotinib; and OR 2·25 (1·92, 2·63) compared to imatinib). Lastly, with regards to infectious diseases (all kinds), no difference was observed comparing the incidence between CML and non-oncologic patients. Although there were previous studies on this topics [7, 10, 11], this is the first time these findings have been shown using real-world data and at a global level instead of local registries or clinical trials. Taken together, these data reinforce the importance of TKI discontinuation whenever possible to avoid further adverse events.

To assess the landscape of TKI treatment throughout the years, we analyzed the survival probability by comparing patients who started treatment between the years 2001–2010 and between 2011–2020. Most records available were from the second time period, with the majority of them belonging to the U.S. cohort. Despite the large cohorts, in order to make fair and balanced comparisons, applying propensity score matching significantly reduced the cohorts to 519 patients for each time period from the Global network (includes all the HCOs from the EMEA and U.S. networks along with HCOs from the Latin America and Asia Pacific regions). Differences seen in the survival probability at the end of the time window or rate of patients with outcomes were due to one cohort having a longer follow-up than the other (since 2001 vs since 2011). The Kaplan–Meier survival analysis showed a perfect overlapping between survival trend in both cohorts, suggesting no change in survival probability after the year 2010 when other TKIs besides Imatinib became available. The median survival was not met during the current follow-up time (Supplementary Fig. 3 and Supplementary Tables 3A, B). Thus, the survival benefit accomplished with the introduction of TKIs at the beginning of the 2000s has remained largely unchanged in the following couple of decades [12], suggesting that the overall impact of using second- and third-generation TKIs has probably had a smaller impact than expected. Then we analyzed whether or not the first line TKI impacted survival within the 10 years after the start of treatment. No difference in mortality was seen in any of the three cohorts comparing survival probability between imatinib and dasatinib or nilotinib, neither comparing dasatinib with nilotinib (Table 1). Although long-term follow-up of dasatinib and nilotinib approval clinical trials focused on response parameters, it was already noted there was no real benefit regarding survival [13, 14]. This was confirmed by a meta-analysis including all TKIs, where no difference in 5-year overall survival was found [15]. In our study, with a relatively short follow-up period (10 years), the use of second-generation TKIs as first-line treatment had no impact on incremental survival.

**Table 1:** Number of patients in each cohort receiving each TKI as the first line of treatment, along with the number and percentage of patients with the outcome (death during treatment).

	Imatinib		Dasatinib		Nilotinib		Imatinib vs Dasatinib		Imatinib vs Nilotinib		Dasastinib vs Nilotinib	
	Total	Dead % (*n*)	Total	Dead % (*n*)	Total	Dead % (*n*)	HR	95% CI	HR	95% CI	HR	95% CI
H12O	44	9,1 (4)	27	11,1 (3)	7	0 (0)	1.2	(0,25–5,84)	–	–	–	–
EMEA	186	24,7 (46)	43	23,3 (10)	35	28,6 (10)	1.29	(0,63–2,68)	1.26	(0,61–2,58)	1.07	(0,42–2,71)
US	2831	23,5 (667)	1842	19,8 (364)	758	20,7 (157)	1.02	(0,89–1,16)	1.1	(0,93–1,31)	1.07	(0,89–1,29)

The hazard ratios comparing survival probability between each TKI evidences no significant differences between them. The same analysis was run using propensity score matching, again with no differences between them.

We also analyzed the survival probability between CML patients (excluding those with second malignancies) and propensity score-matched non-oncologic patients. In the H12O cohort, 32 patients from each cohort were compared, with two deaths in the CML cohort and 5 in the non-oncology patients (*p* = 0·23). In EMEA cohort, there were 35 deaths in 147 CML patients versus 29 in 147 non-oncology patients (*p* = 0·40). The U.S. cohort also showed slightly better survival in non-oncologic patients but without statistical significance, 284 deaths out of 1852 CML patients versus 251 deaths out of 1852 non-oncologic patients, with an HR 1·095 (0·92–1·29), *p* = 0·295 (Supplementary Fig. 4).

There are some study limitations worth mentioning. Most data available in TriNetX comes from structured EHR, so it is possible that some patients that meet the inclusion criteria have been excluded due to their data being recorded in free text, and therefore, not available in TriNetX. Furthermore, EHR data is subject to data entry errors and data gaps. Mortality data may be incomplete in some organizations or reported with some delay. To try to minimize this, only patients with recorded death or with a visit to their HCO since January 1, 2021 were included in the analysis and we excluded from the survival analysis the data network where out-of-hospital deaths are not tracked consistently. Although data were not centrally curated, H12O CML dedicated datasets were used as a reference for controlling the quality of CML data in all cohorts. On the other hand, most queries were checked on three different cohorts, with different sample sizes and different geographical settings. This, along with the use of propensity score matching, should provide additional strength to the results obtained from the study. To conclude, this large-scale study based on real-world data confirms the previous finding of CML patients reaching a similar survival as the general population but also raises a major concern about the development of comorbidities and poorer quality of life after the diagnosis of CML. Therefore, the study reinforces the power of real-world studies based on global federated health research networks to confirm the results of clinical trials.

## Supplementary information

Supplementary figures and tables

## Data Availability

The data that support the findings of this study are available from TriNetX, LLC but third-party restrictions apply to the availability of these data. The data were used under license for this study with restrictions that do not allow for the data to be redistributed or made publicly available. However, for accredited researchers, the TriNetX data is available for licensing at TriNetX, LLC. Data access may require a data-sharing agreement and may incur data access fees.
