# Supplementary material for: Outcomes and patterns of treatment in chronic myeloid leukemia, a global perspective based on a real-world data global network
Source: Blood Cancer J. 2022 Jun 24;12(6):94. doi: 10.1038/s41408-022-00692-8 (PMC9232604; doi:10.1038/s41408-022-00692-8)
Supplement: Supplementary file 1 — Supplementary figures and tables [file 41408_2022_692_MOESM1_ESM.pdf]

Supplementary figures and tables

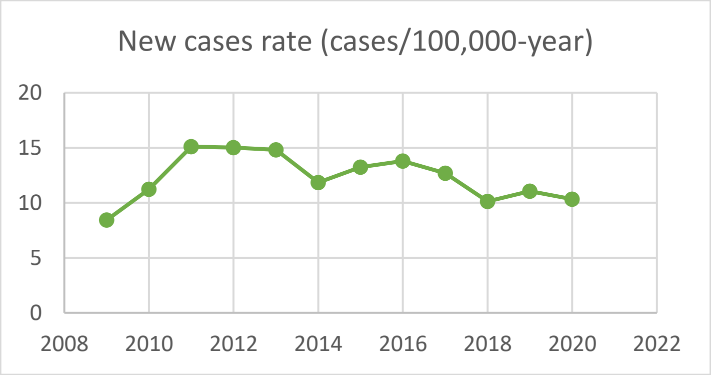

Supplementary figure 1A. New cases rate from the US Collaborative Network. The reference population comes from non-CML patient records in the queried institutions, so this data cannot be compared with regular epidemiologic CML studies.

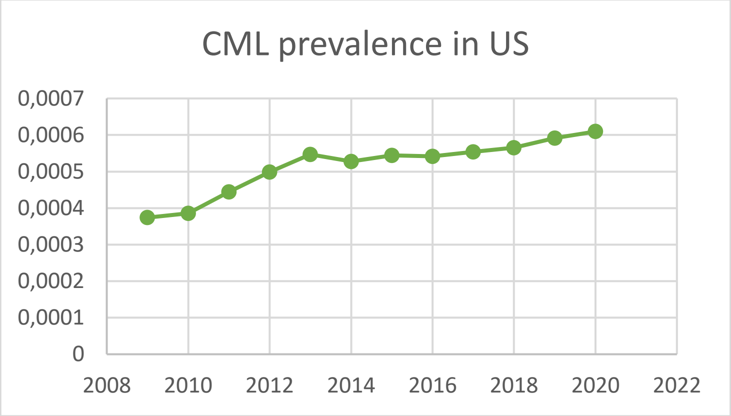

Supplementary figure 1B. Prevalence in the US Collaborative Network

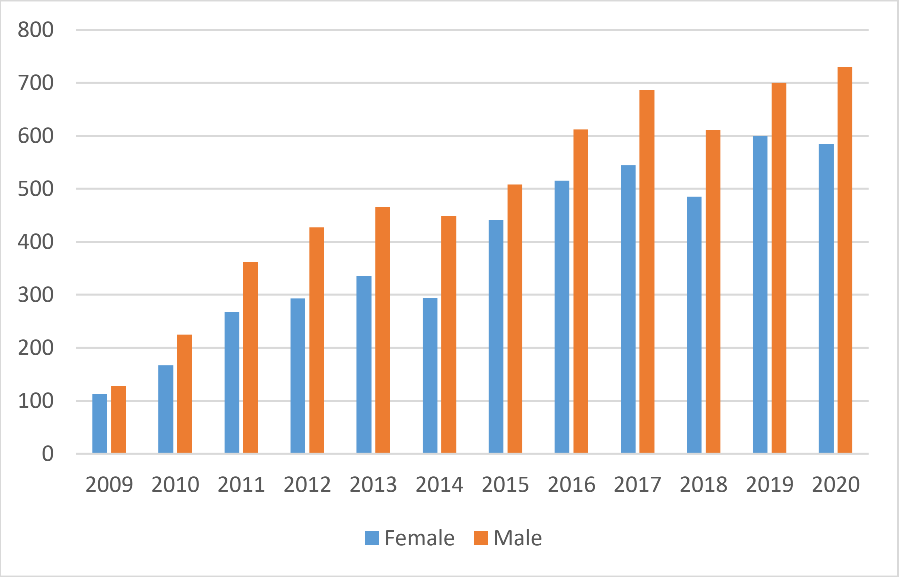

Supplementary figure 1C. Incident Cases from the US Collaborative Network

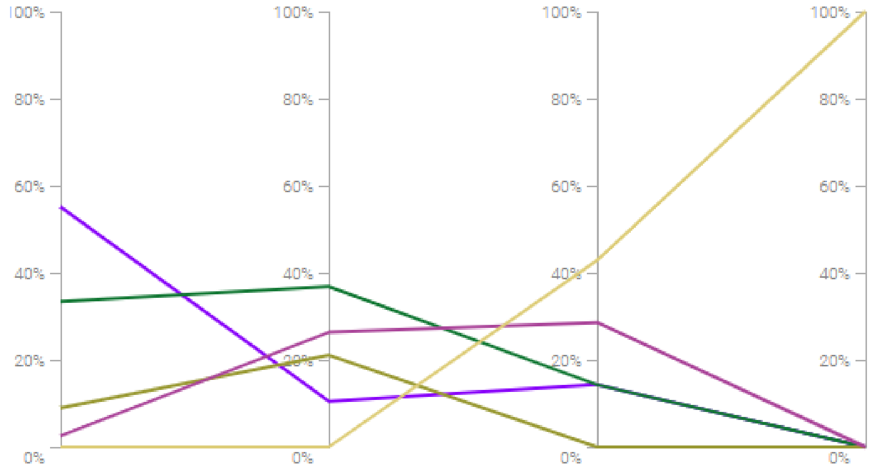

Supplementary figure 2A. **Treatment pathway in H12O.** Imatinib (purple), Dasatinb (green), Nilotinib (light green), Bosutinib (pink), Ponatinib (yellow).

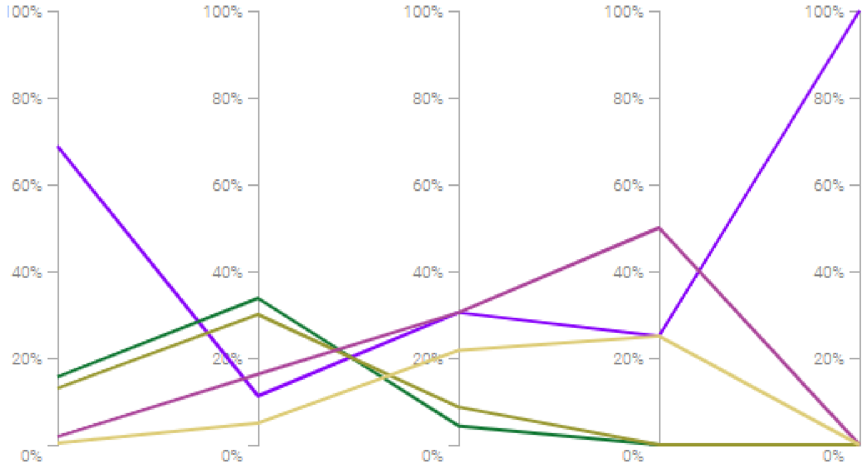

Supplementary figure 2B. **Treatment pathway in EMEA.** Imatinib (purple), Dasatinb (green), Nilotinib (light green), Bosutinib (pink), Ponatinib (yellow).

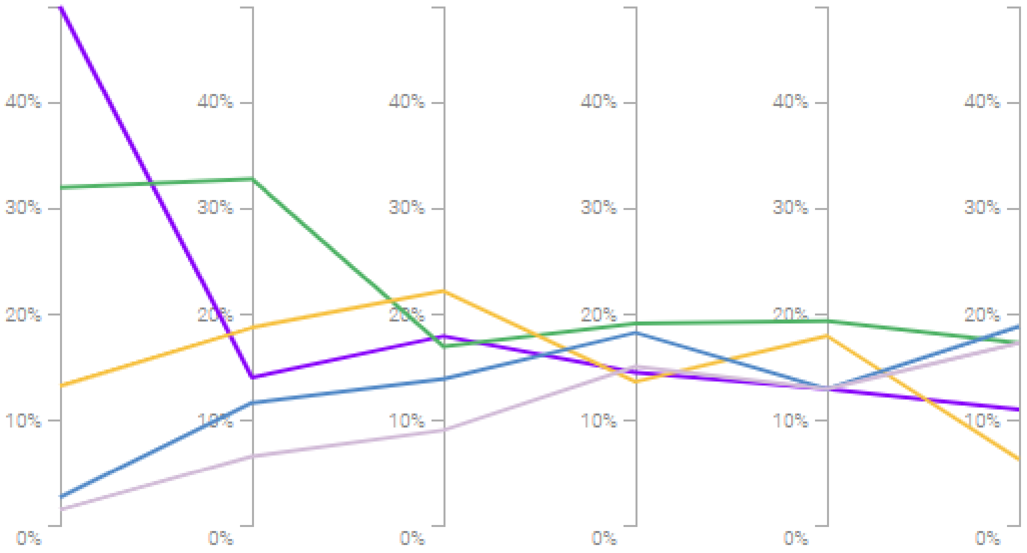

Supplementary Figure 2C. **Treatment pathway in the US.** Imatinib (purple), Dasatinb (green), Nilotinib (light green), Bosutinib (blue), Ponatinib (grey).

Supplementary figure 2. **Treatment pathways in H12O (2C) in EMEA (2B) and the US (2C).** Analyzing the proportion of patients receiving each TKI as first-line of treatment and the frequency of subsequent switching to other TKIs (Figure 2, exact counts in supplementary table 1), imatinib was the most used TKI as first line treatment in all cohorts, and dasatinib the most used as second-line. H12O and the US both had a greater proportion of patients in dasatinib compared to nilotinib, while EMEA summed a similar amount of patients on both second generation TKI throughout all lines of treatment. Usage of ponatinib increased almost after every switching, with its use ranging 10-20% between third and sixth line.

Treatment	LOT 1: Patients	LOT 1: Percent	LOT 2: Patients	LOT 2: Percent	LOT 3: Patients	LOT 3: Percent	LOT 4: Patients	LOT 4: Percent
imatinib	43	55.13%	2	10.53%	1	14.29%	0	0.00%
dasatinib	26	33.33%	7	36.84%	1	14.29%	0	0.00%
nilotinib	7	8.97%	4	21.05%	0	0.00%	0	0.00%
bosutinib	2	2.56%	5	26.32%	2	28.57%	0	0.00%
ponatinib	0	0.00%	0	0.00%	3	42.86%	1	100.00%

Supplementary table 1A, Exact counts and percentages of patients receiving each TKI in H12O.

Treatment	LOT 1: Patients	LOT 1: Percent	LOT 2: Patients	LOT 2: Percent	LOT 3: Patients	LOT 3: Percent	LOT 4: Patients	LOT 4: Percent	LOT 5: Patients	LOT 5: Percent
imatinib	185	69.03%	9	11.69%	7	31.82%	1	25.00%	1	100.00%
dasatinib	42	15.67%	27	35.06%	1	4.55%	0	0.00%	0	0.00%
nilotinib	35	13.06%	24	31.17%	2	9.09%	0	0.00%	0	0.00%
bosutinib	5	1.87%	13	16.88%	7	31.82%	2	50.00%	0	0.00%
ponatinib	1	0.37%	4	5.19%	5	22.73%	1	25.00%	0	0.00%

Supplementary table 1B, Exact counts and percentages of patients receiving each TKI in the EMEA.

Treatment	LOT 1: Patients	LOT 1: Percent	LOT 2: Patients	LOT 2: Percent	LOT 3: Patients	LOT 3: Percent	LOT 4: Patients	LOT 4: Percent	LOT 5: Patients	LOT 5: Percent	LOT 6: Patients	LOT 6: Percent
imatinib	2823	49.95%	274	16.74%	151	22.40%	50	17.99%	18	16.98%	7	15.56%
dasatinib	1838	32.52%	642	39.22%	143	21.22%	66	23.74%	27	25.47%	11	24.44%
nilotinib	757	13.39%	366	22.36%	187	27.74%	47	16.91%	25	23.58%	4	8.89%
bosutinib	151	2.67%	227	13.87%	117	17.36%	63	22.66%	18	16.98%	12	26.67%
ponatinib	83	1.47%	128	7.82%	76	11.28%	52	18.71%	18	16.98%	11	24.44%

Supplementary table 1C, Exact counts and percentages of patients receiving each TKI in the US.

Cohort 1 and cohort 2 patient count before and after propensity score matching			
	Cohort	Patient count before matching	Patient count after matching
**H12O**	1 - CML treated	80	57
	2 - non CML	124,838	57
**EMEA**	1 - CML treated	271	247
	2 - non CML	829,425	247
**US**	1 - US CML treated visit	5,781	5,249
	2 - US non CML visit	15,044,411	5,249

				Characteristics before PSM				Characteristics after PSM			
	Cohort			Mean ± SD	Patients	% of Cohort	P-Value	Mean ± SD	Patients	% of Cohort	P-Value
**H12O**	1	Age	Current Age	56.2 +/- 16.0	57	100%	0.858	56.2 +/- 16.0	57	100%	1
	2			56.8 +/- 25.0	92,262	100%		56.2 +/- 16.0	57	100%	
	1	F	Female		22	38.6%	0.022		22	38.6%	1
	2				49,577	53.7%			22	38.6%	
	1	M	Male		35	61.4%	0.022		35	61.4%	1
	2				42,685	46.3%			35	61.4%	
**EMEA**	1	Age	Current Age	64.8 +/- 17.9	247	100%	<0.001	64.8 +/- 17.9	247	100%	1
	2			55.0 +/- 24.4	767,492	100%		64.8 +/- 17.9	247	100%	
	1	F	Female		98	39.7%	<0.001		98	39.7%	1
	2				427,823	55.7%			98	39.7%	
	1	M	Male		149	60.3%	<0.001		149	60.3%	1
	2				339,257	44.2%			149	60.3%	
**US**	1	Age	Current Age	61.0 +/- 16.8	5,249	100%	<0.001	61.0 +/- 16.8	5,249	100%	1
	2			47.8 +/- 24.7	14,051,890	100%		61.0 +/- 16.8	5,249	100%	
	1	F	Female		2,348	44.7%	<0.001		2,348	44.7%	1
	2				7,959,899	56.6%			2,348	44.7%	
	1	M	Male		2,9	55.2%	<0.001		2,9	55.2%	1
	2				6,089,718	43.3%			2,9	55.2%	

Supplementary table 2. **CML (1) and non- CML (2) cohorts in H12O, EMEA and US.**

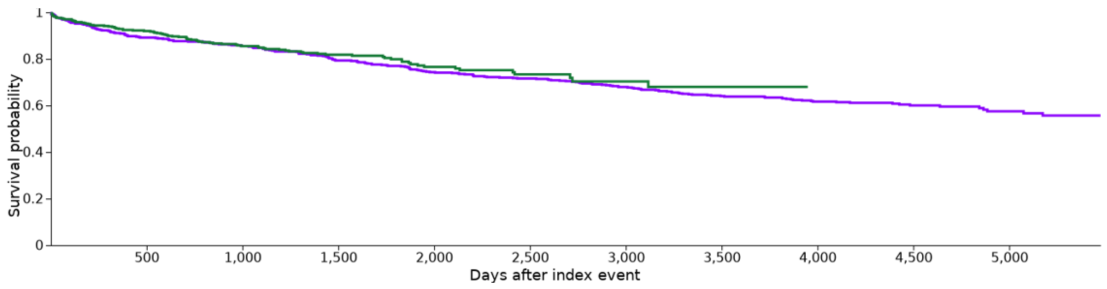

Supplementary figure 3. Survival probability of CML patients from the Global Network treated between 2001-2010 (purple) and between 2011-2020 (green).

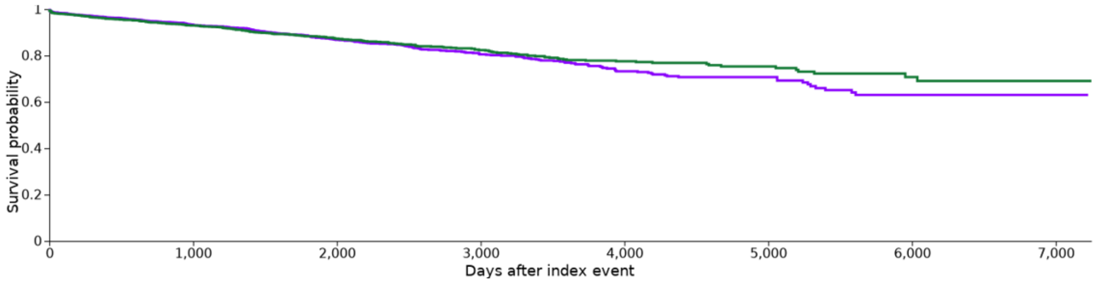

Supplementary figure 4. Kaplan Meier analysis CML (purple) vs non-oncologic patients (green) in the US cohort

1 Mortality

Risk analysis

Cohort		Patients in cohort	Patients with outcome	Risk
1	Global CML treated started in 2001 - 2010	519	208	0.401
2	Global CML treated start in 2011-2020	519	93	0.179

		95% CI	z	p
Risk Difference	0.222	(0.168, 0.275)	7.866	0.000
Risk Ratio	2.237	(1.809, 2.765)	N/A	N/A
Odds Ratio	3.064	(2.304, 4.073)	N/A	N/A

Supplementary table 3A. Risk analysis of CML pre and post 2010 with propensity score matching

Kaplan - Meier survival analysis				
Cohort	Patients in cohort	Patients with outcome	Median survival (days)	Survival probability at end of time window
1 Global CML treated started in 2001 - 2010	519	208	--	55.72%
2 Global CML treated start in 2011-2020	519	93	--	68.01%

	χ^2	df	p
**Log-Rank Test**	0.651	1	0.420

	Hazard Ratio	95% CI	χ^2	df	p
**Hazard Ratio and Proportionality**	1.113	(0.858, 1.443)	0.000	1	0.985

Supplementary table 3B. Kaplan Meier analysis of CML pre and post 2010 with propensity score matching
